# Supplementary material for: The Influence of the Matrix on the Apatite-Forming Ability of Calcium Containing Polydimethylsiloxane-Based Cements for Endodontics
Source: Molecules. 2022 Sep 6;27(18):5750. doi: 10.3390/molecules27185750 (PMC9504520; doi:10.3390/molecules27185750)
Supplement: Supplementary file 1 [file molecules-27-05750-s001.zip › molecules-1868108-SI.pdf]

*Supplementary Material*

# The influence of the matrix on the apatite forming ability of calcium containing polydimethylsiloxane-based cements for endodontics

*Paola Taddei, Michele Di Foggia, Fausto, Zamparini, Carlo Prati, Maria Giovanna Gandolfi*

**Table S1.** Wavenumbers and assignments [20-25] of the main IR bands detected in the spectra of the commercial cements (orange and white pastes, just mixed sealers).

| Wavenumbers / $cm^{-1}$ | Assignments |
|---|---|
| 3055 | =CH stretching |
| 2963 | $CH_3$ antisymmetric stretching |
| 2905 | $CH_3$ symmetric stretching |
| 2160 | Si-H stretching |
| 1600 | C=C stretching |
| 1450 | $CH_3$ antisymmetric bending |
| 1411 | $CH_3$ symmetric bending |
| 1258 | $CH_3$ symmetric deformation |
| 1078 | antisymmetric Si-O-Si stretching |
| 1010 | symmetric Si-O-Si stretching |
| 930 | non-bridging oxygen, Si–$O_{NBO}$ stretching mode |
| 910 | Si-H bending |
| 866 | $CH_3$ symmetric rocking |
| 790 | $CH_3$ antisymmetric rocking, Si-C antisymmetric stretching |
| 740 | Si-C symmetric stretching, Si-C antisymmetric bending, Si-O bending, monoclinic $ZrO_2$ |
| 572 | monoclinic $ZrO_2$ |
| 490 | monoclinic $ZrO_2$ |
| 450 | monoclinic $ZrO_2$ |

**Table S2.** Wavenumbers and assignments [21,24,27-29] of the main Raman bands detected in the FT-Raman spectra of the commercial cements (orange and white pastes, just mixed sealers).

| Wavenumbers / $cm^{-1}$ | Assignments |
|---|---|
| 3055 | =CH stretching |
| 2967 | $CH_3$ antisymmetric stretching |
| 2908 | $CH_3$ symmetric stretching |
| 2870 | $CH_3$ symmetric stretching |
| 1670 | C=C stretching guttapercha |
| 1600 | C=C stretching |
| 1411 | $CH_3$ symmetric bending |
| 1265 | $CH_3$ symmetric deformation |
| 1080 | Si-O-Si stretching of $SiO_4$ tetrahedra |
| 960-940 | $PO_4$ stretching |
| 861 | $CH_3$ symmetric rocking |
| 790 | $CH_3$ antisymmetric rocking, Si-C antisymmetric stretching |
| 755 | Si-C symmetric stretching, Si-C antisymmetric bending, Si-O bending, monoclinic $ZrO_2$ |
| 710 | Si-C symmetric stretching |
| 690 | Si-$CH_3$ symmetric rocking |
| 638 | monoclinic $ZrO_2$ |
| 616 | monoclinic $ZrO_2$ |
| 559 | monoclinic $ZrO_2$ |
| 537 | monoclinic $ZrO_2$ |
| 500 | monoclinic $ZrO_2$ |
| 476 | monoclinic $ZrO_2$ |
| 435 | ZnO |
| 382 | monoclinic $ZrO_2$ |
| 347 | monoclinic $ZrO_2$ |
| 334 | monoclinic $ZrO_2$ |
| 306 | monoclinic $ZrO_2$ |
| 221 | monoclinic $ZrO_2$ |
| 191 | monoclinic $ZrO_2$ |
| 179 | monoclinic $ZrO_2$ |

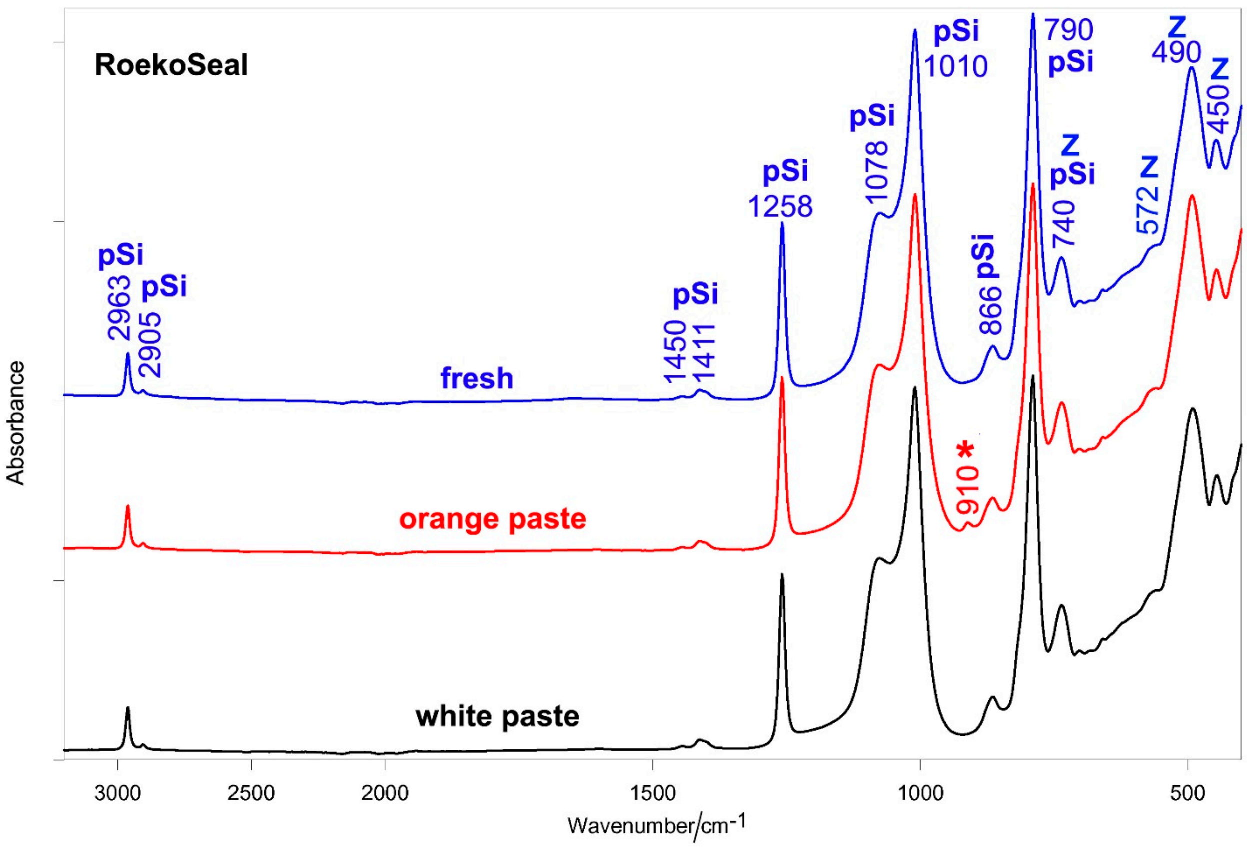


**Figure S1**. Average IR spectrum of fresh (i.e. just mixed) RoekoSeal; the spectra of its white and orange components are reported for comparison. The spectra are normalized to the absorbance of the 2963 $cm^{-1}$ band. The bands assignable to polydimethylsiloxane (pSi) and monoclinic zirconia (Z) are indicated together with those specifically assigned to Si-H bonds (*).

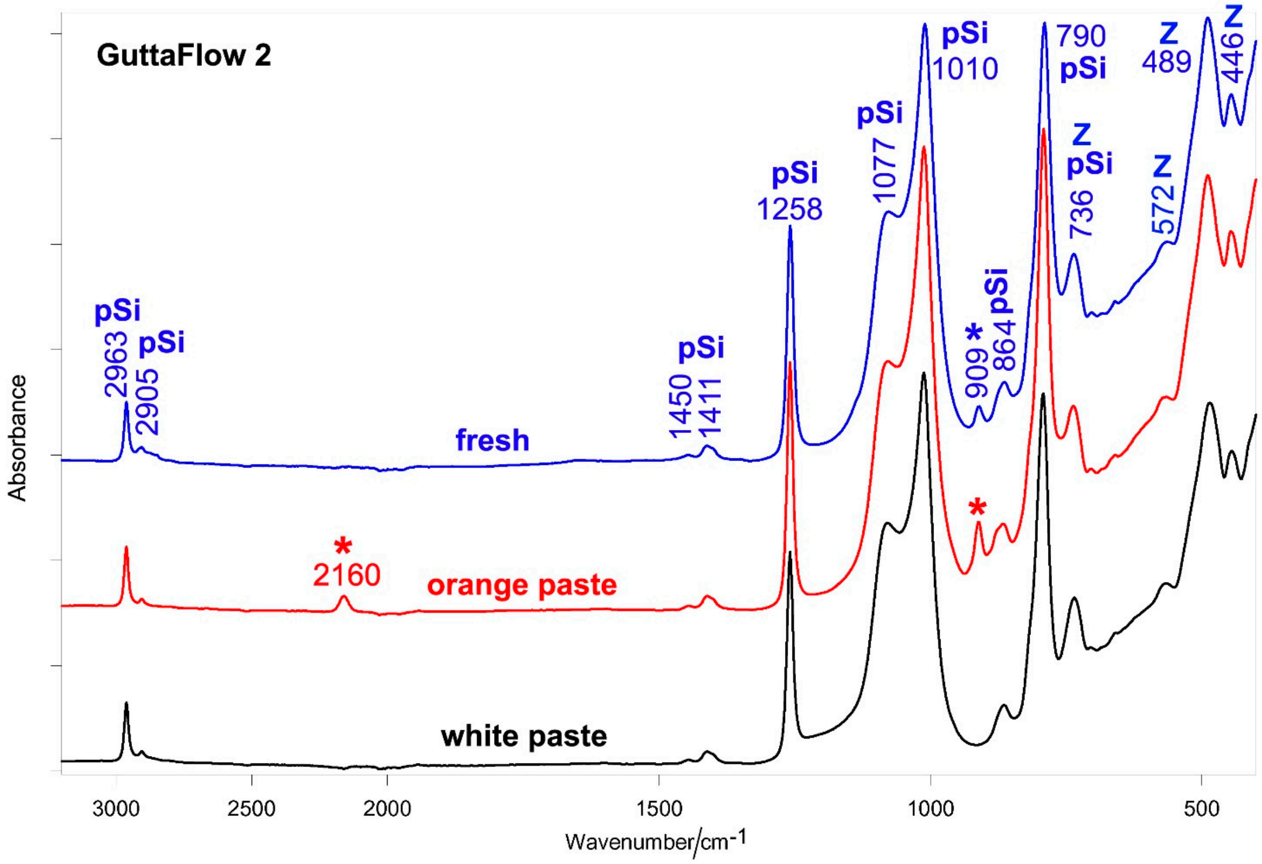


**Figure S2**. Average IR spectrum of fresh (i.e. just mixed) GuttaFlow 2; the spectra of its white and orange components are reported for comparison. The spectra are normalized to the absorbance of the 2963 cm$^{-1}$ band. The bands assignable to polydimethylsiloxane (pSi) and monoclinic zirconia (Z) are indicated together with those specifically assigned to Si-H bonds (*).

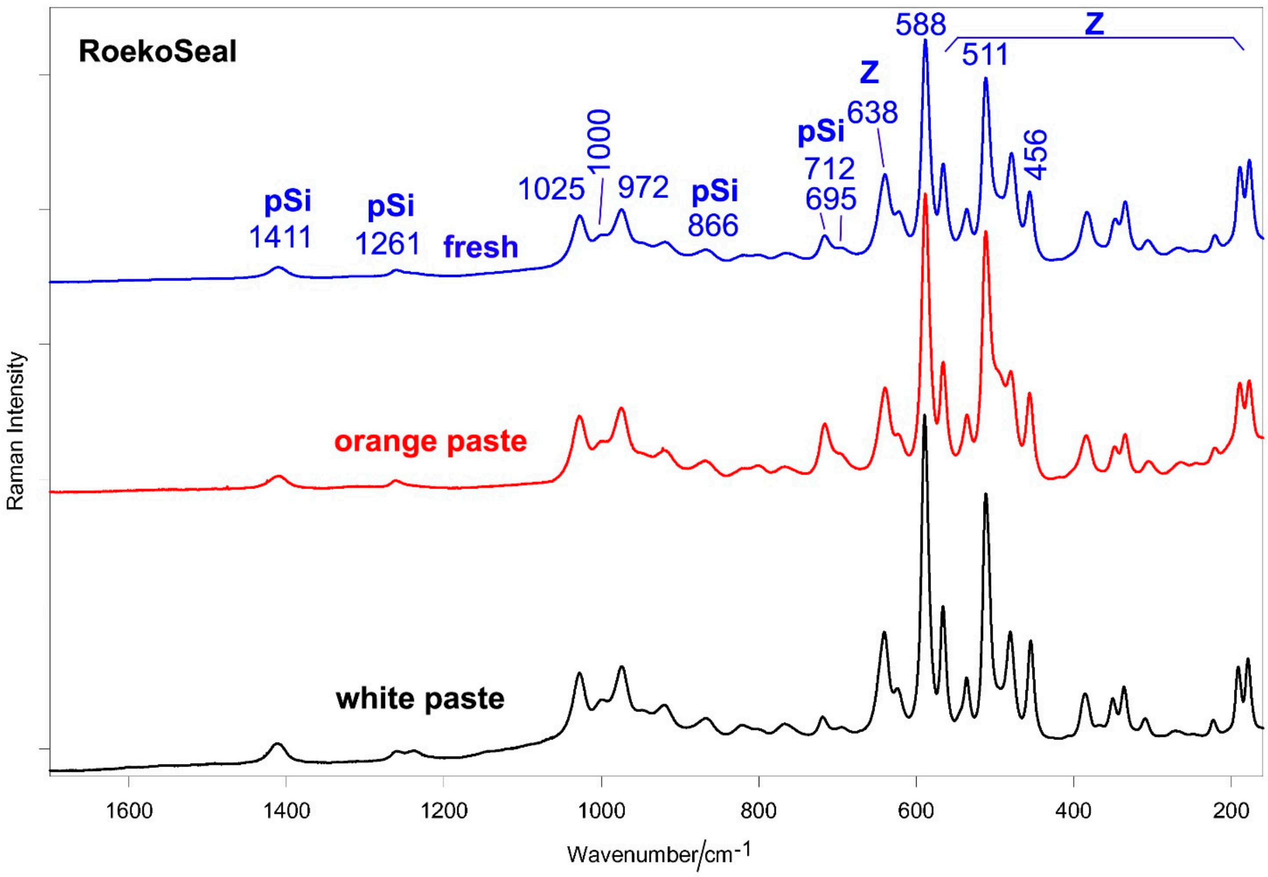


**Figure S3**. Average micro-Raman spectra of fresh (i.e. just mixed) RoekoSeal, its white and orange pastes. The spectra are normalized to the intensity of the 638 $cm^{-1}$ band. The bands assignable to monoclinic zirconia (Z) and polydimethylsiloxane (pSi) are indicated.

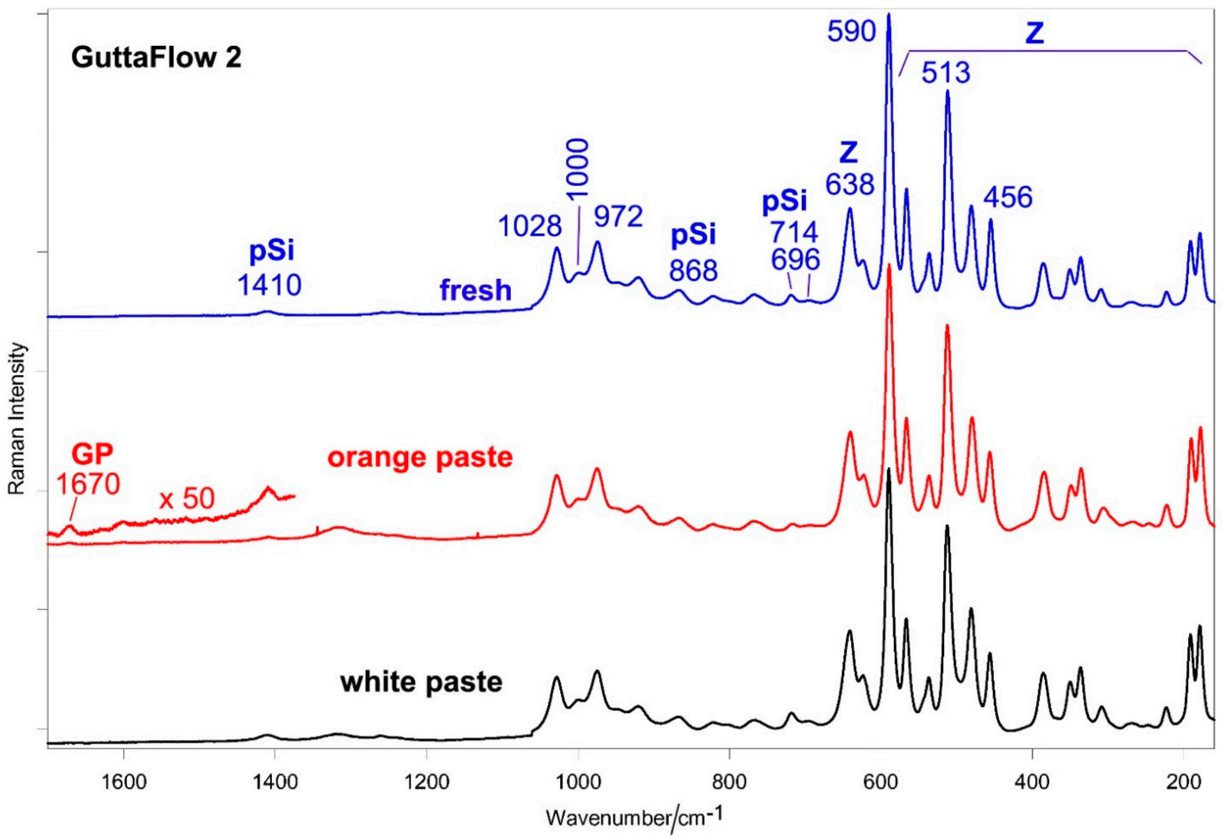


**Figure S4**. Average micro-Raman spectra of fresh (i.e. just mixed) GuttaFlow 2, its white and orange pastes. The spectra are normalized to the intensity of the 638 $cm^{-1}$ band. The bands assignable to monoclinic zirconia (Z), polydimethylsiloxane (pSi) and guttapercha (GP) are indicated.

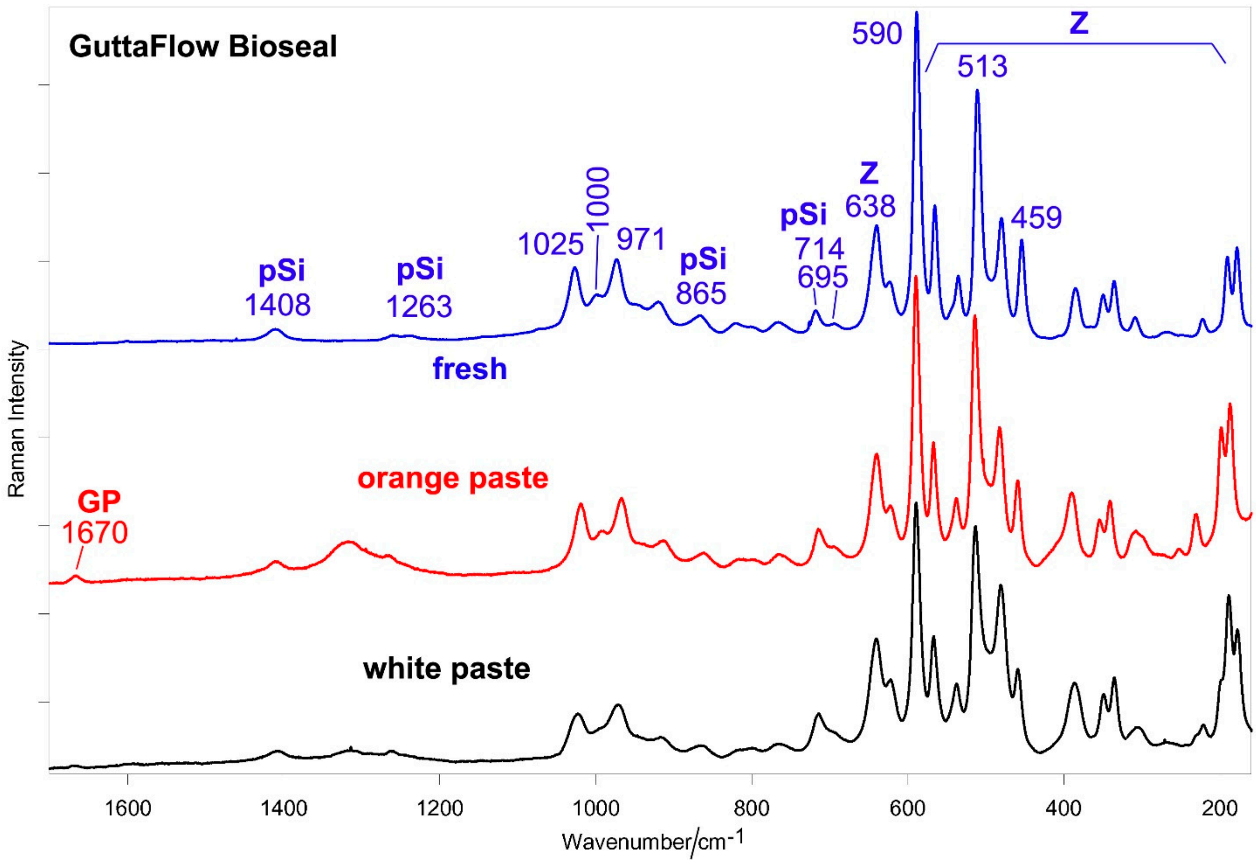


**Figure S5**. Average micro-Raman spectra of fresh (i.e. just mixed) GuttaFlow Bioseal, its white and orange pastes. The spectra are normalized to the intensity of the 638 cm-1 band. The bands assignable to monoclinic zirconia (Z), polydimethylsiloxane (pSi) and guttapercha (GP) are indicated.

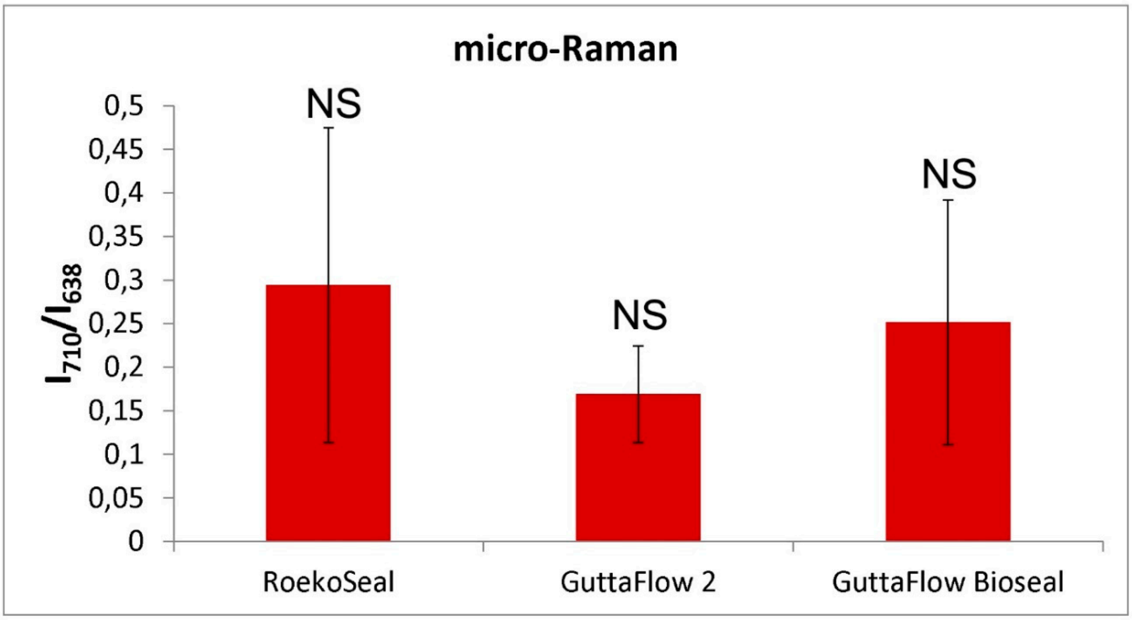


**Figure S6**. $I_{710}/I_{638}$ intensity ratio (average ± standard deviation) as calculated from the micro-Raman spectra of fresh commercial sealers under study. No statistically significant differences were observed between the values (NS, $P > 0.05$).

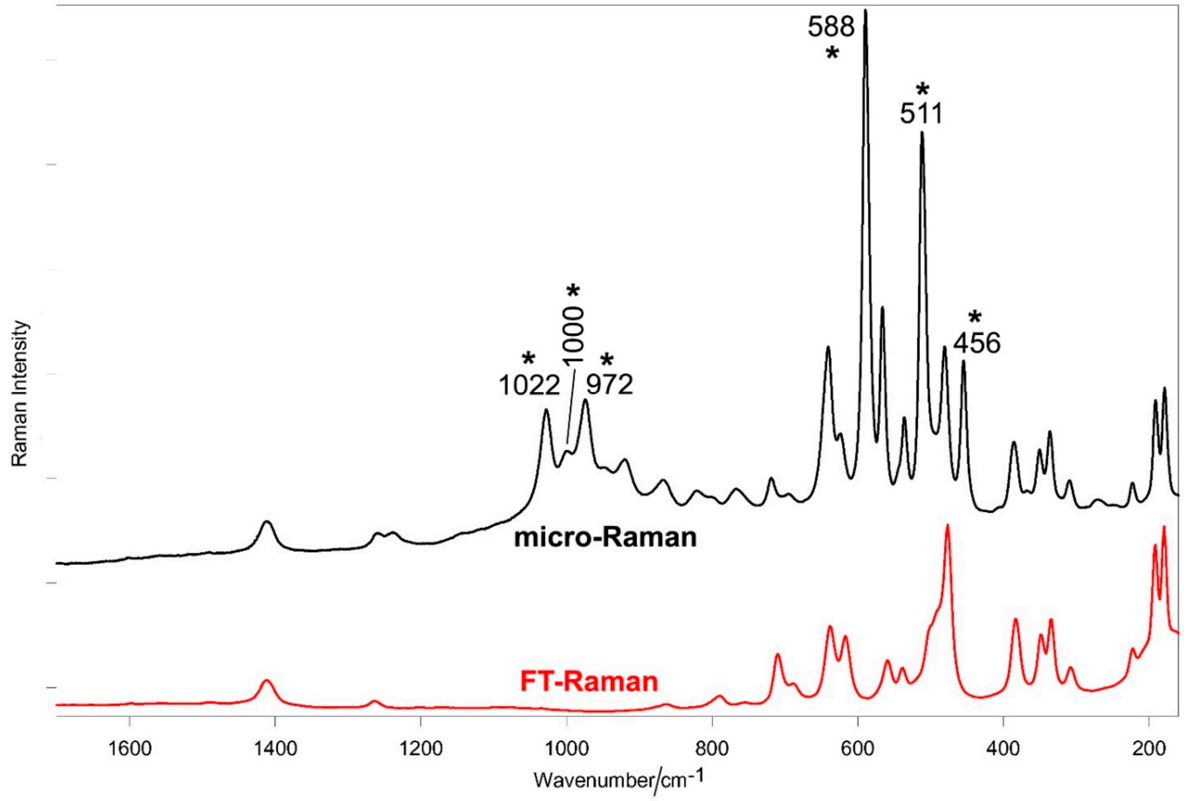


**Figure S7**. Average FT-Raman and micro-Raman spectra of white paste of RoekoSeal. The bands observed only in the micro-Raman spectrum are indicated with an asterisk.

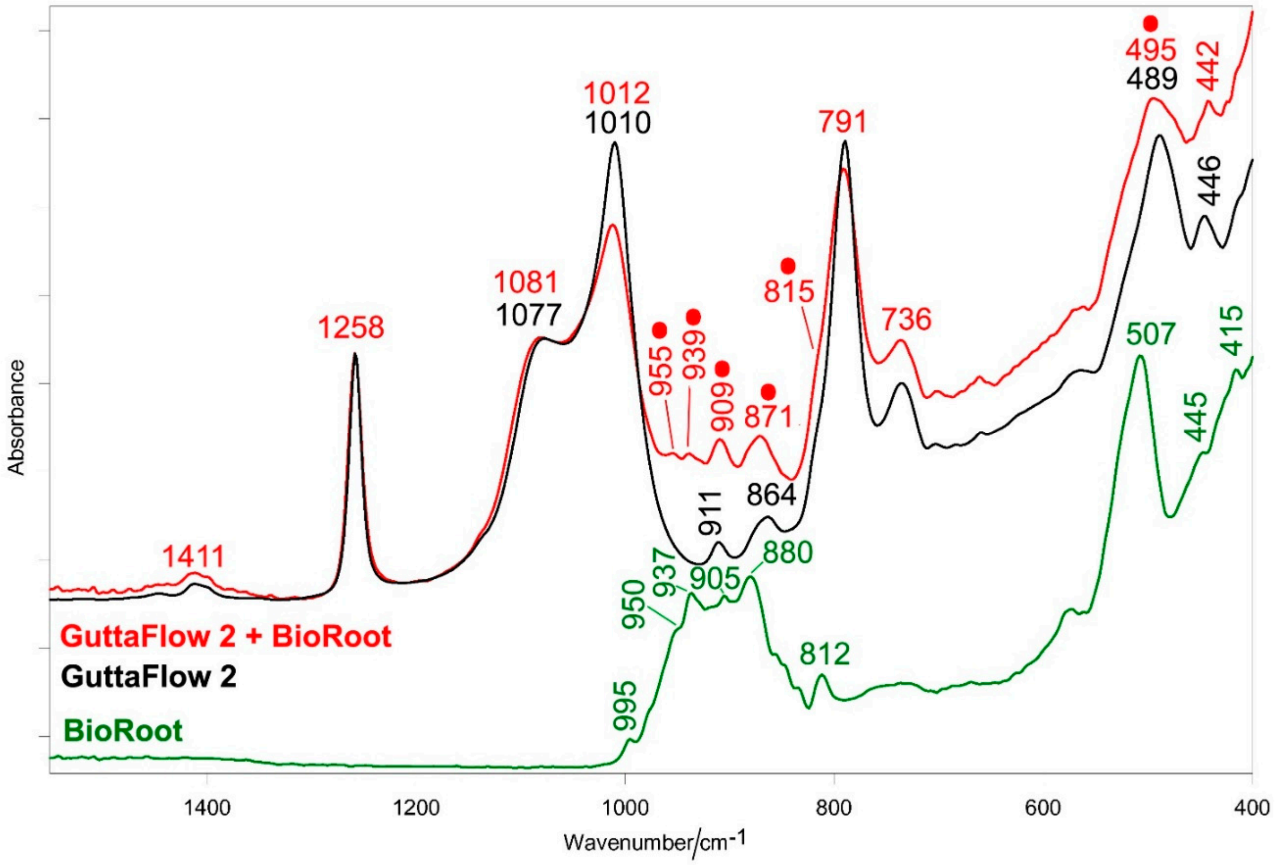


**Figure S8.** Average IR spectra of fresh GuttaFlow 2 + 20% BioRoot RCS and Guttaflow 2, normalized to the absorbance of the 1258 $cm^{-1}$ band. The spectrum of BioRoot RCS is reported for comparison; spectral features assignable to it are indicated with a circle.

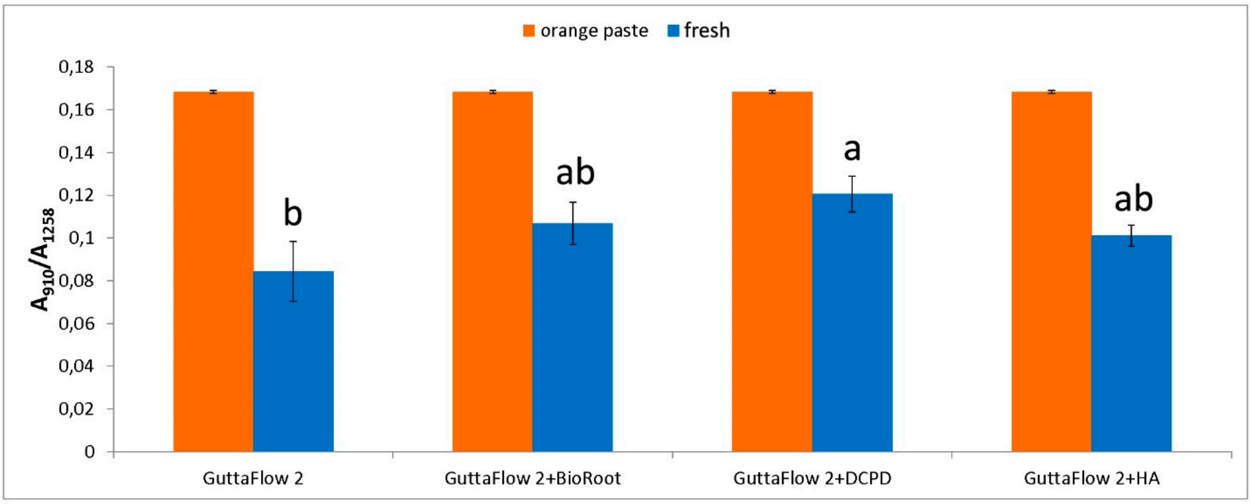


**Figure S9.** $A_{910}/A_{1258}$ absorbance ratio (average ± standard deviation) as calculated from the IR spectra of the orange paste and fresh samples of the doped sealers under study (GuttaFlow 2 is reported for comparison). Different letters represent statistically significant differences ($P < 0.05$) between the fresh samples.

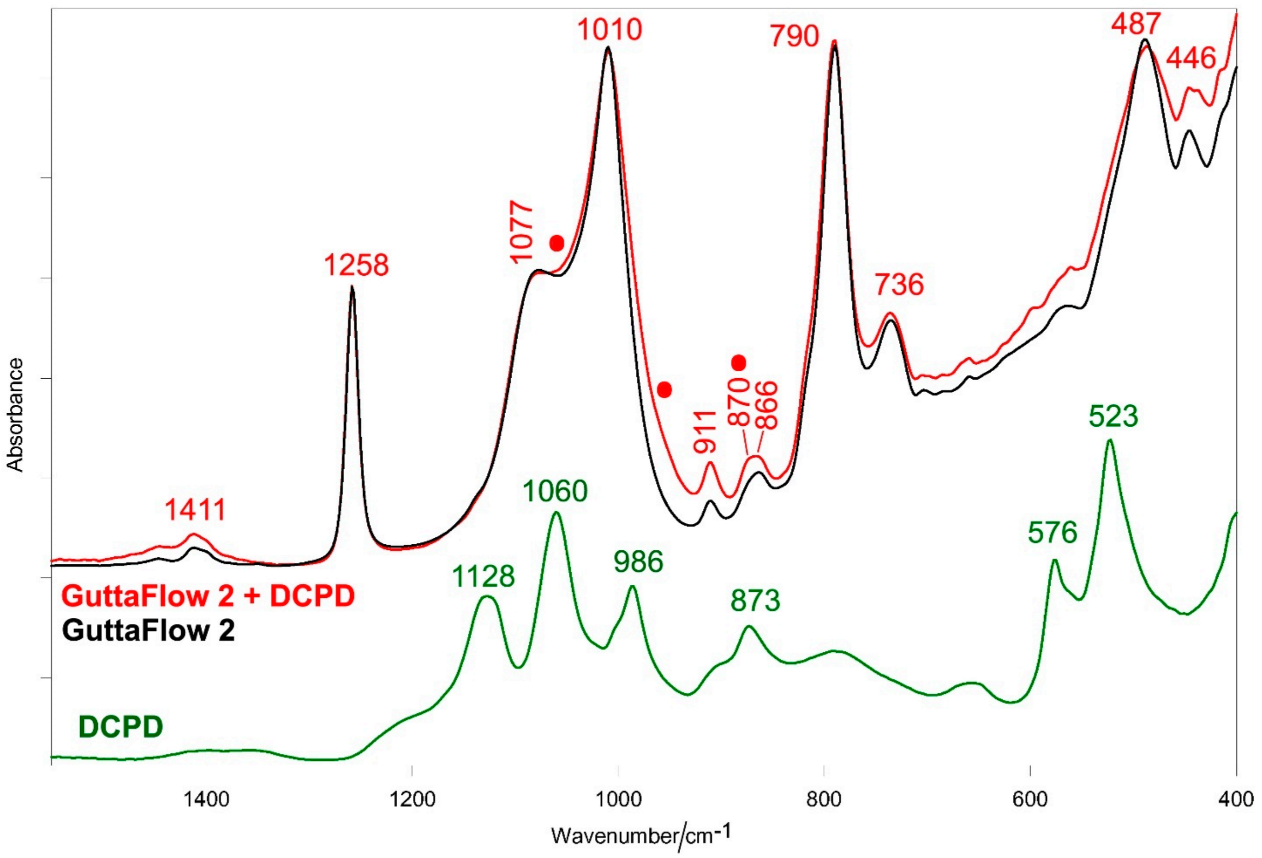


**Figure S10**. Average IR spectra of fresh GuttaFlow 2 + 20% DCPD and Guttaflow 2, normalized to the absorbance of the 1258 $cm^{-1}$ band. The spectrum of DCPD is reported for comparison; spectral features assignable to it are indicated with a circle.

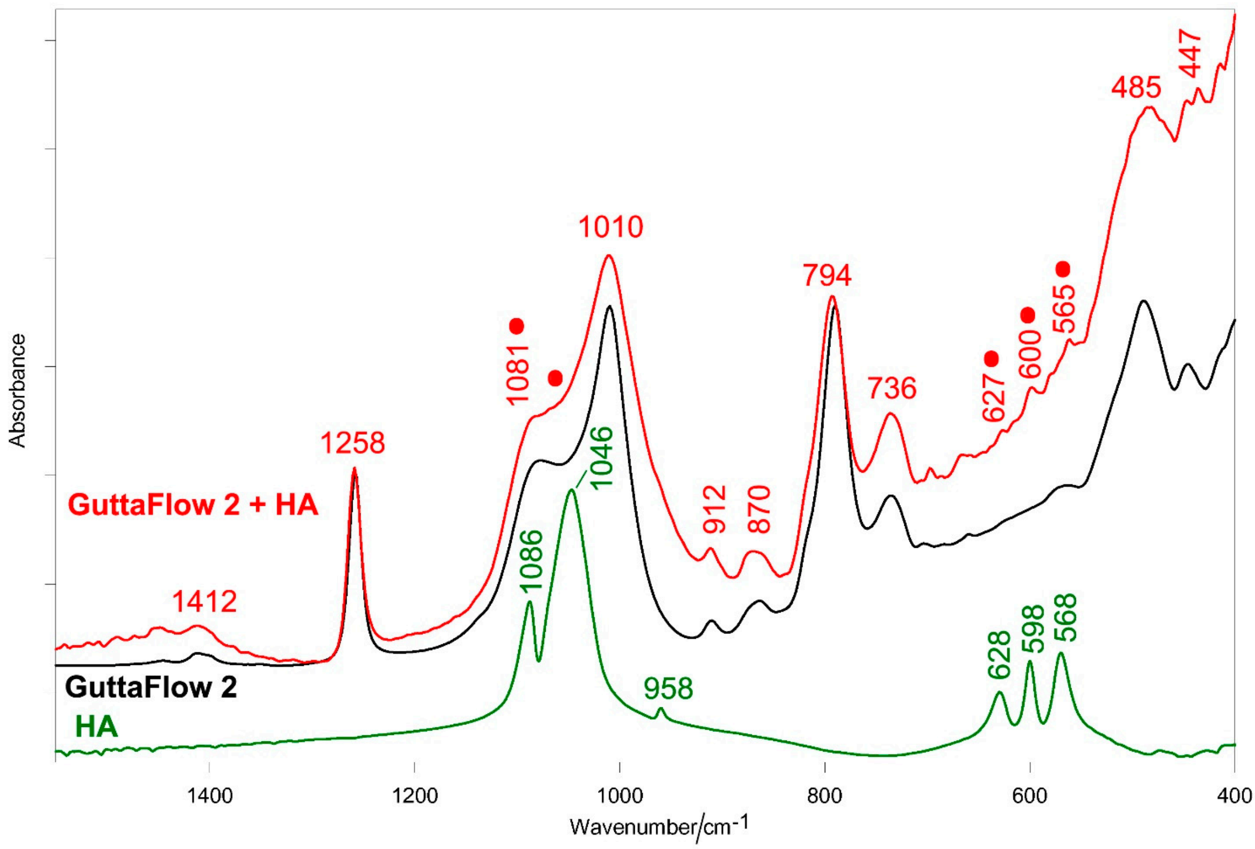


**Figure S11.** Average IR spectra of fresh GuttaFlow 2 + 20% HA and Guttaflow 2, normalized to the absorbance of the 1258 $cm^{-1}$ band. The spectrum of HA is reported for comparison; spectral features assignable to it are indicated with a circle.

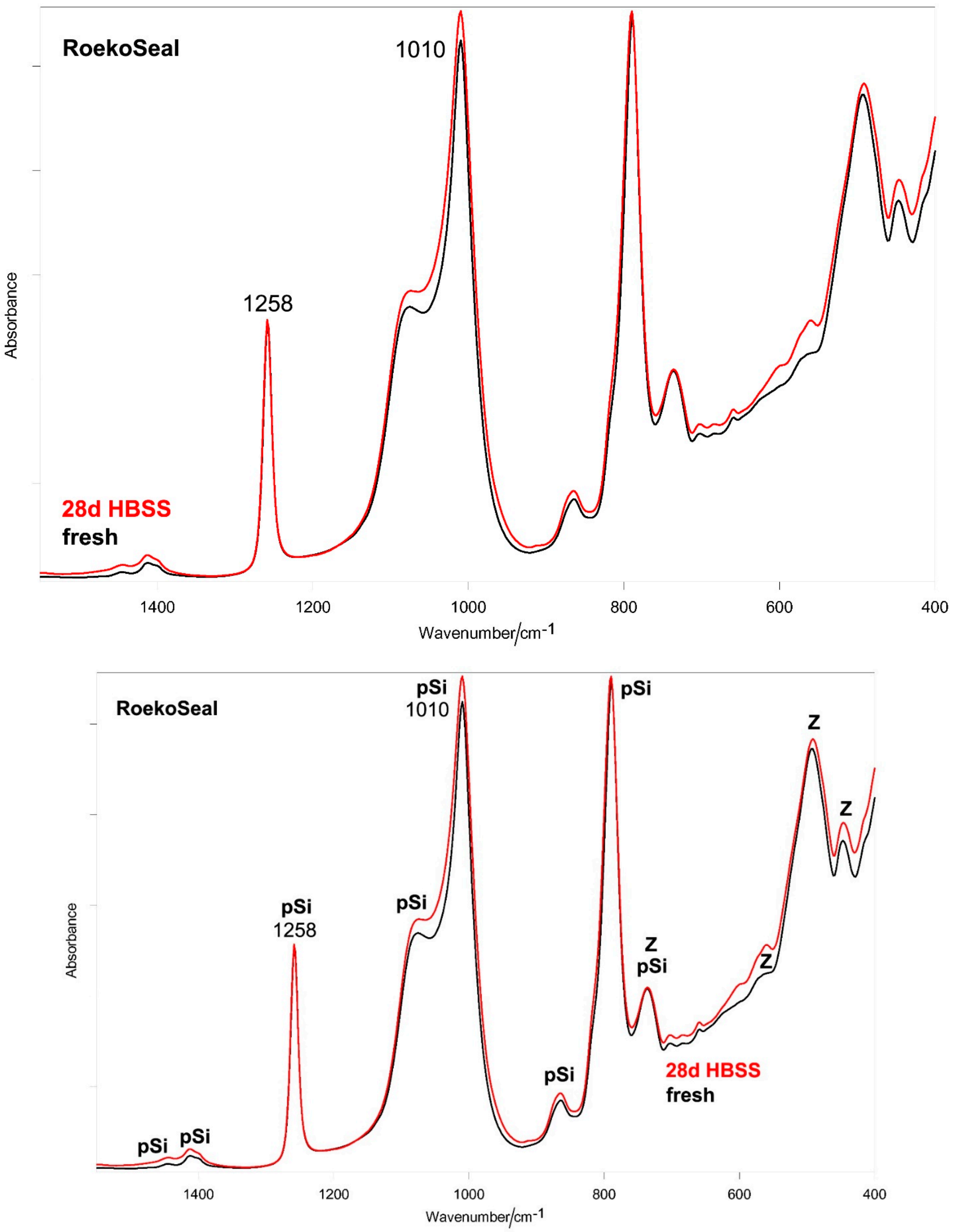


**Figure S12.** Average IR spectra recorded on the surface of RoekoSeal before (i.e. fresh) and after ageing in HBSS for 28 days. The spectra are normalized to the absorbance of the 1258 $cm^{-1}$ band. The bands assignable to polydimethylsiloxane (pSi) and monoclinic zirconia (Z) are indicated.

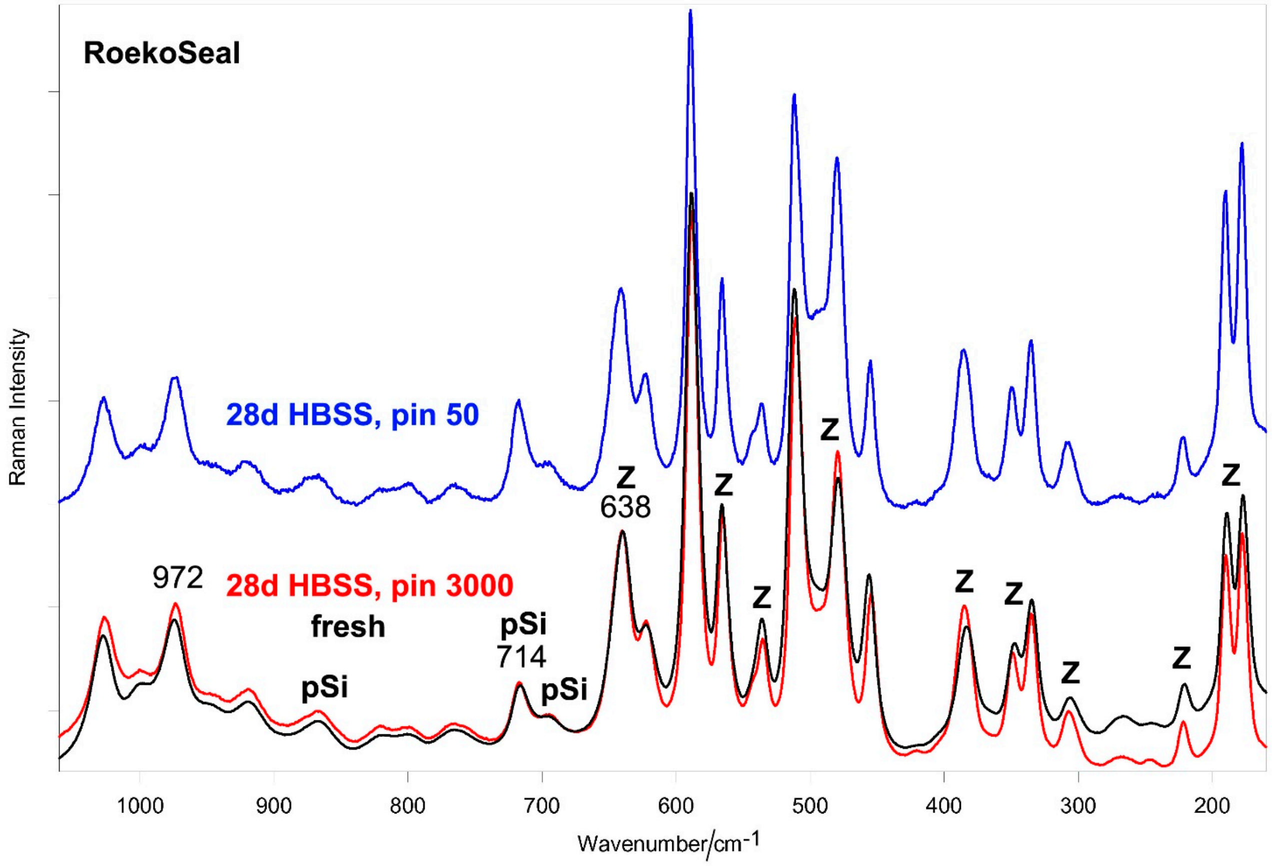


**Figure S13.** Average micro-Raman spectra recorded on the surface of RoekoSeal before (i.e. fresh) and after ageing in HBSS for 28 days. The spectra are normalized to the intensity of the 638 $cm^{-1}$ band. The spectra on the aged sample were recorded using pinholes of 3000 µm (pin 3000) and 50 µm (pin 50). The bands assignable to monoclinic zirconia (Z) and polydimethylsiloxane (pSi) are indicated.

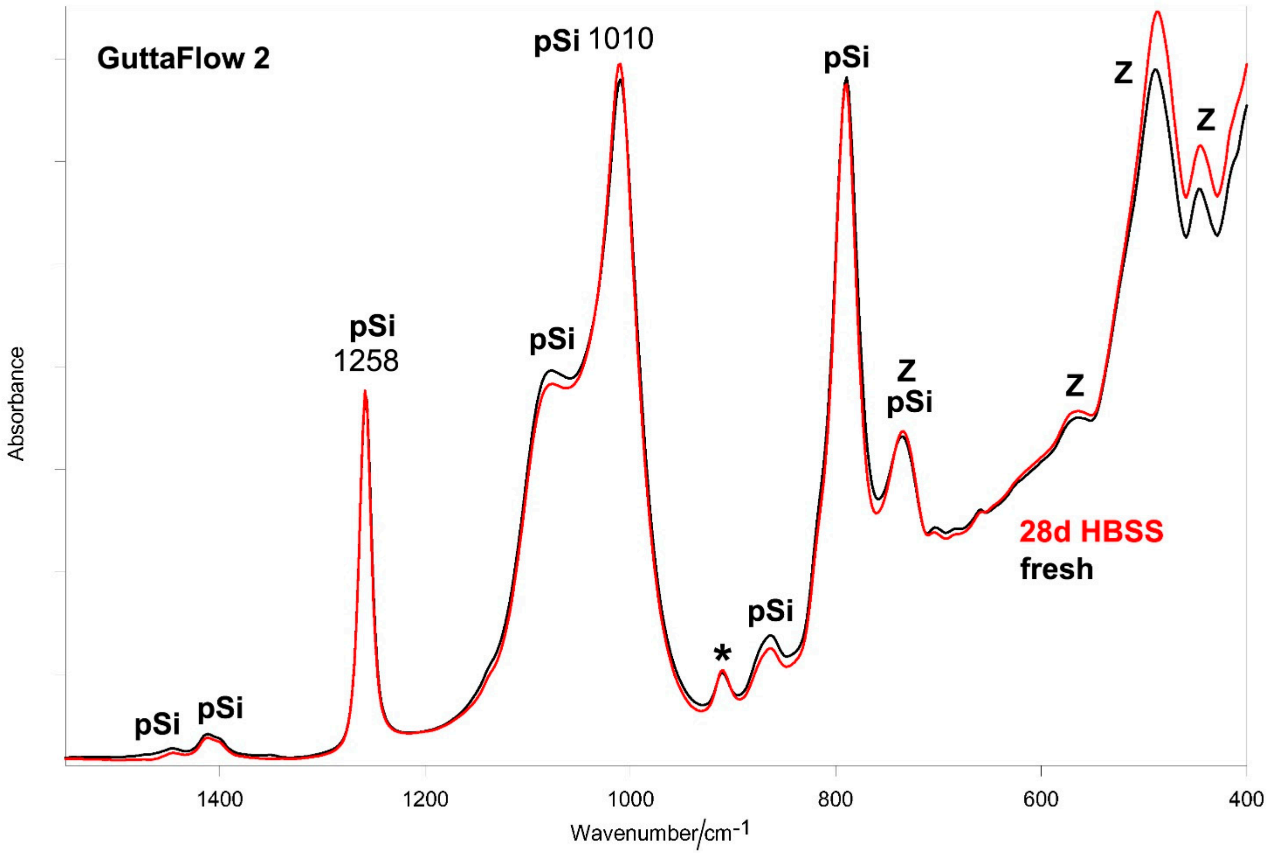


**Figure S14.** Average IR spectra recorded on the surface of GuttaFlow 2 before (i.e. fresh) and after ageing in HBSS for 28 days. The spectra are normalized to the absorbance of the 1258 $cm^{-1}$ band. The bands assignable to polydimethylsiloxane (pSi) and monoclinic zirconia (Z) are indicated together with those specifically assigned to unreacted Si-H bonds (*).

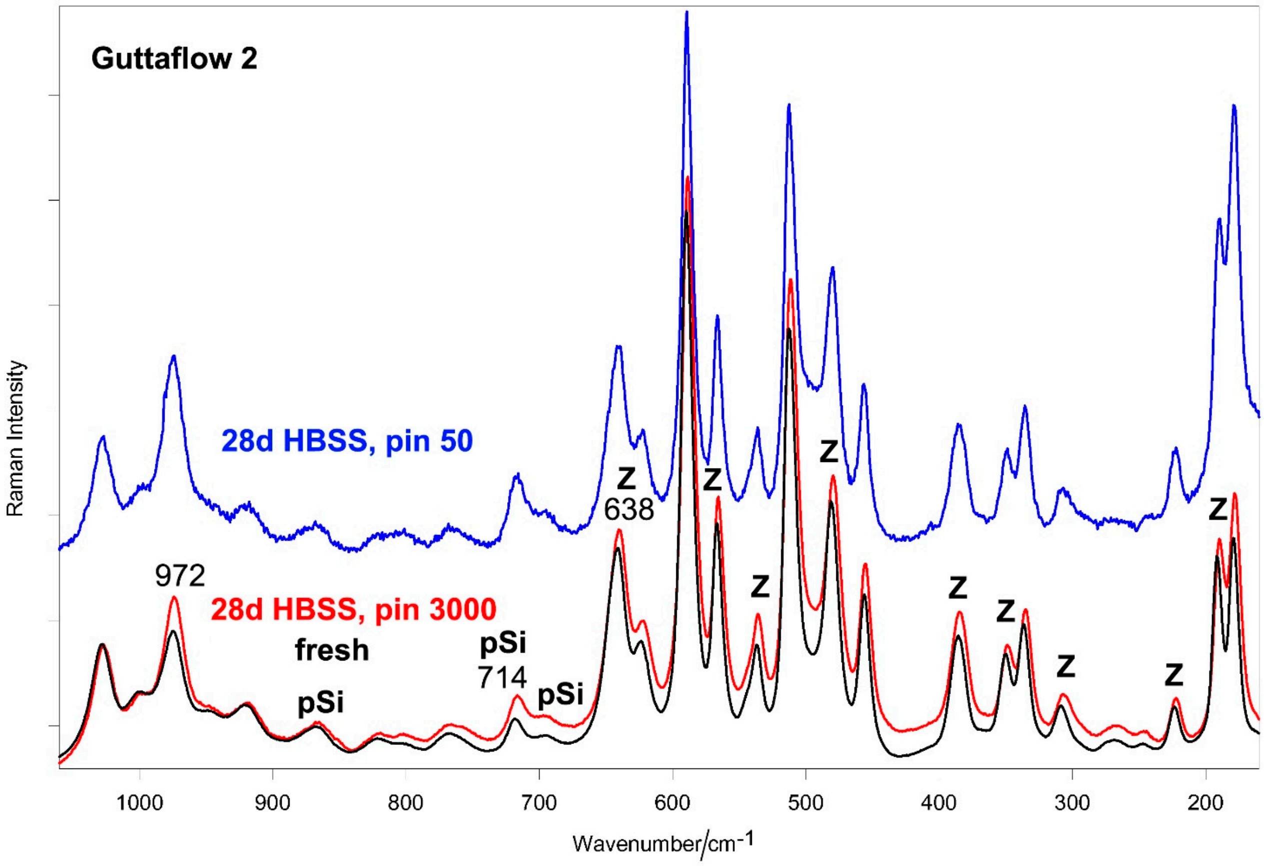


**Figure S15.** Average micro-Raman spectra recorded on the surface of GuttaFlow 2 before (i.e. fresh) and after ageing in HBSS for 28 days. The spectra on the aged sample were recorded using pinholes of 3000 µm (pin 3000) and 50 µm (pin 50). The bands assignable to monoclinic zirconia (Z) and polydimethylsiloxane (pSi) are indicated.

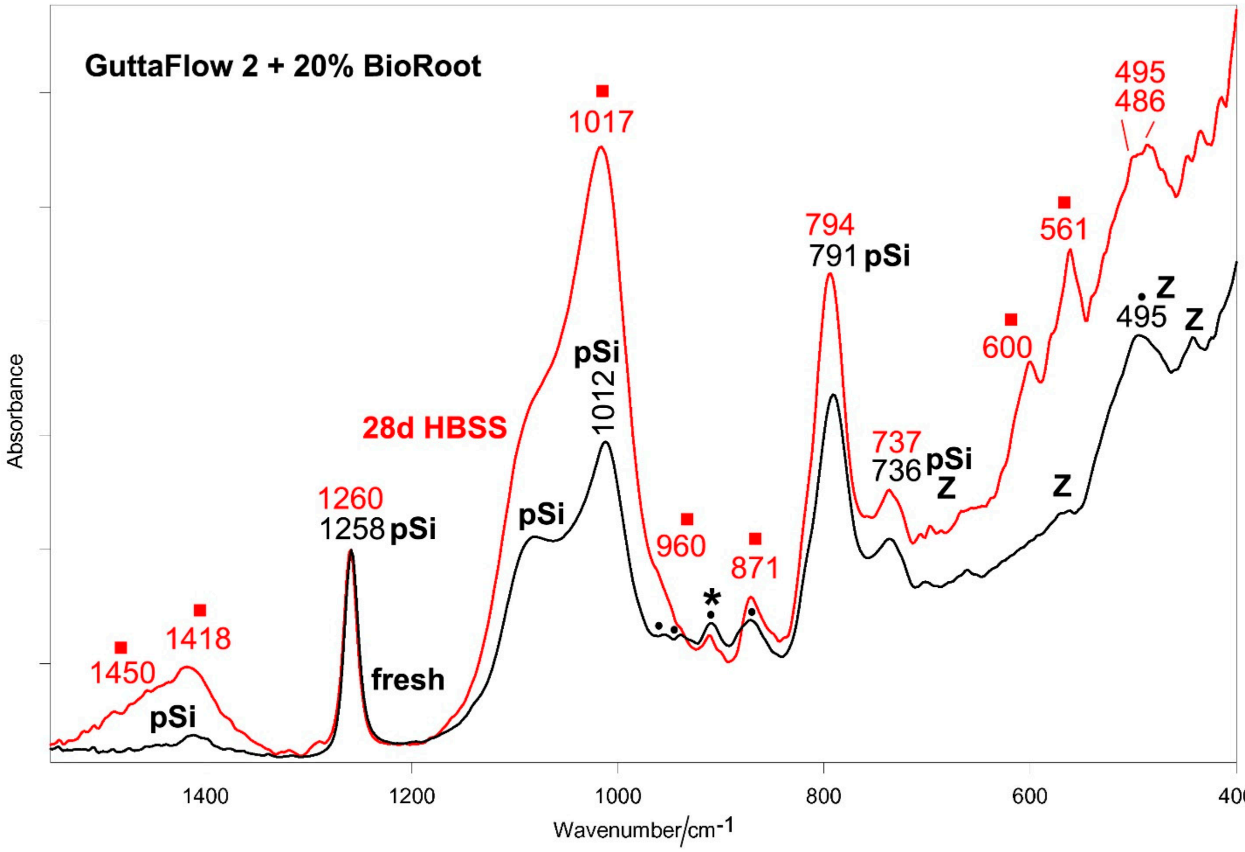


**Figure S16.** Average IR spectra recorded on the surface of GuttaFlow 2 + 20% BioRoot RCS, before (i.e., fresh) and after ageing in HBSS for 28 days. The spectra are normalized to the absorbance of the 1258 cm$^{-1}$ band. The bands assignable to BioRoot RCS doping mineralizing agent (●) and B-type carbonated apatite (■) are indicated together with those of polydimethylsiloxane (pSi), monoclinic zirconia (Z) and unreacted Si-H bonds (*).

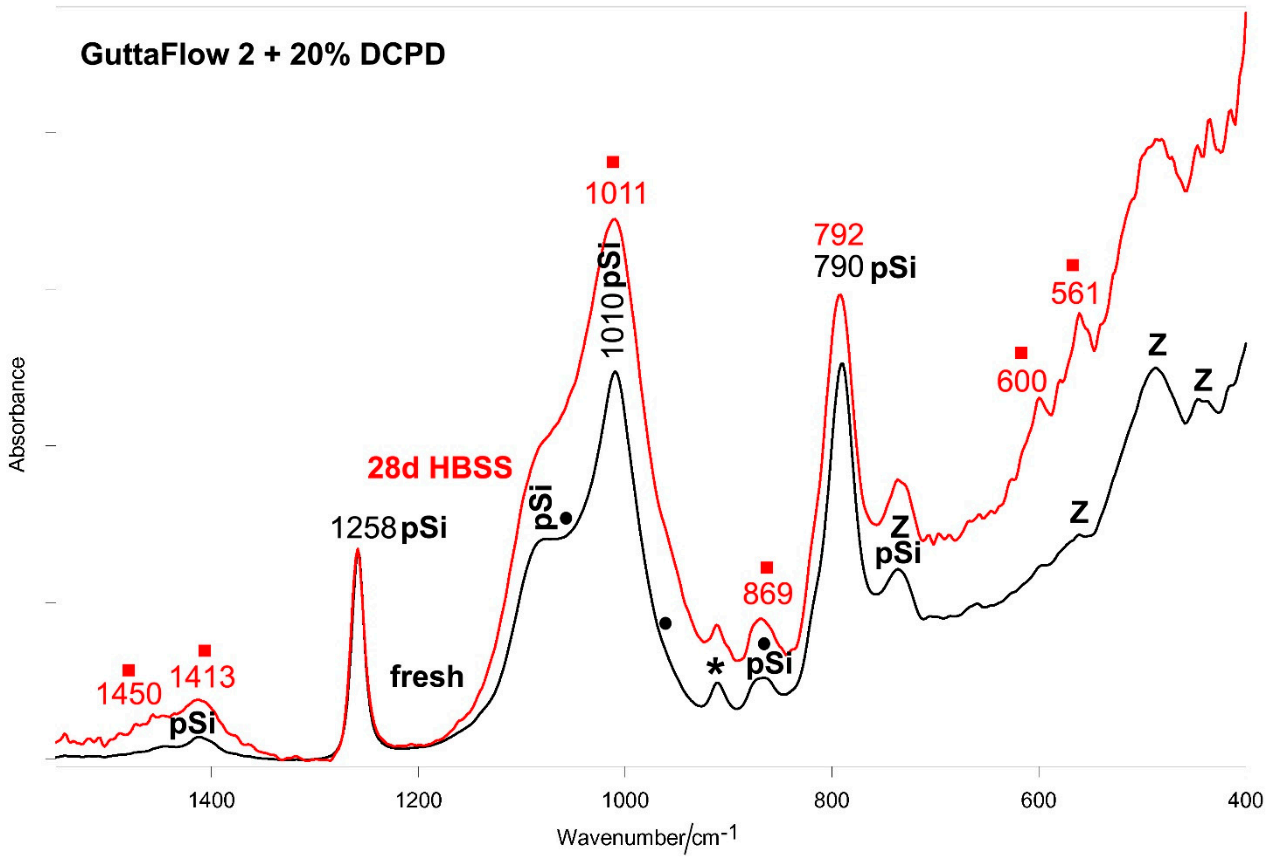


**Figure S17.** Average IR spectra recorded on the surface of GuttaFlow 2 + 20% DCPD, before (i.e., fresh) and after ageing in HBSS for 28 days. The spectra are normalized to the absorbance of the 1258 cm$^{-1}$ band. The bands assignable to DCPD doping mineralizing agent (●) and B-type carbonated apatite (■) are indicated together with those of polydimethylsiloxane (pSi), monoclinic zirconia (Z) and unreacted Si-H bonds (*).

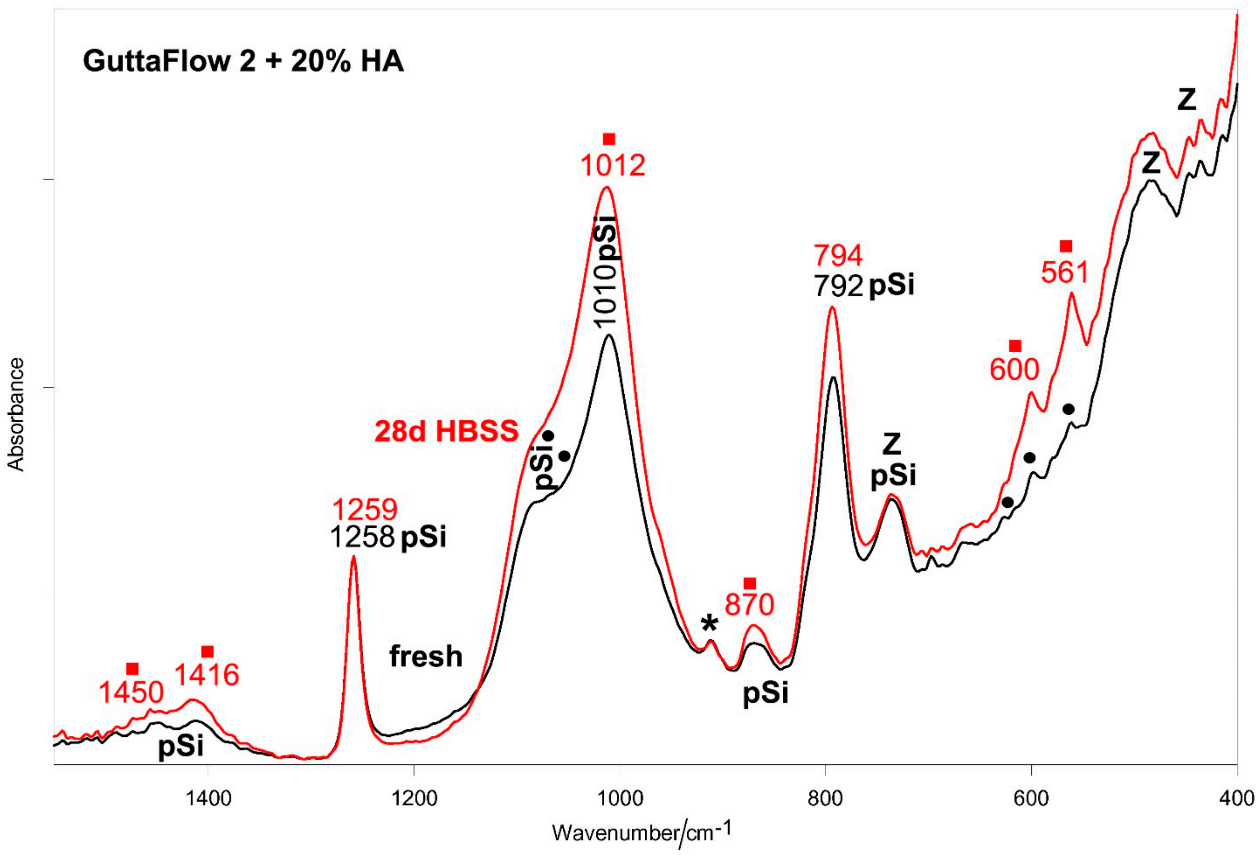


**Figure S18.** Average IR spectra recorded on the surface of GuttaFlow 2 + 20% HA, before (i.e., fresh) and after ageing in HBSS for 28 days. The spectra are normalized to the absorbance of the 1258 $cm^{-1}$ band. The bands assignable to HA doping mineralizing agent (●) and B-type carbonated apatite (■) are indicated together with those of polydimethylsiloxane (pSi), monoclinic zirconia (Z) and unreacted Si-H bonds (*).

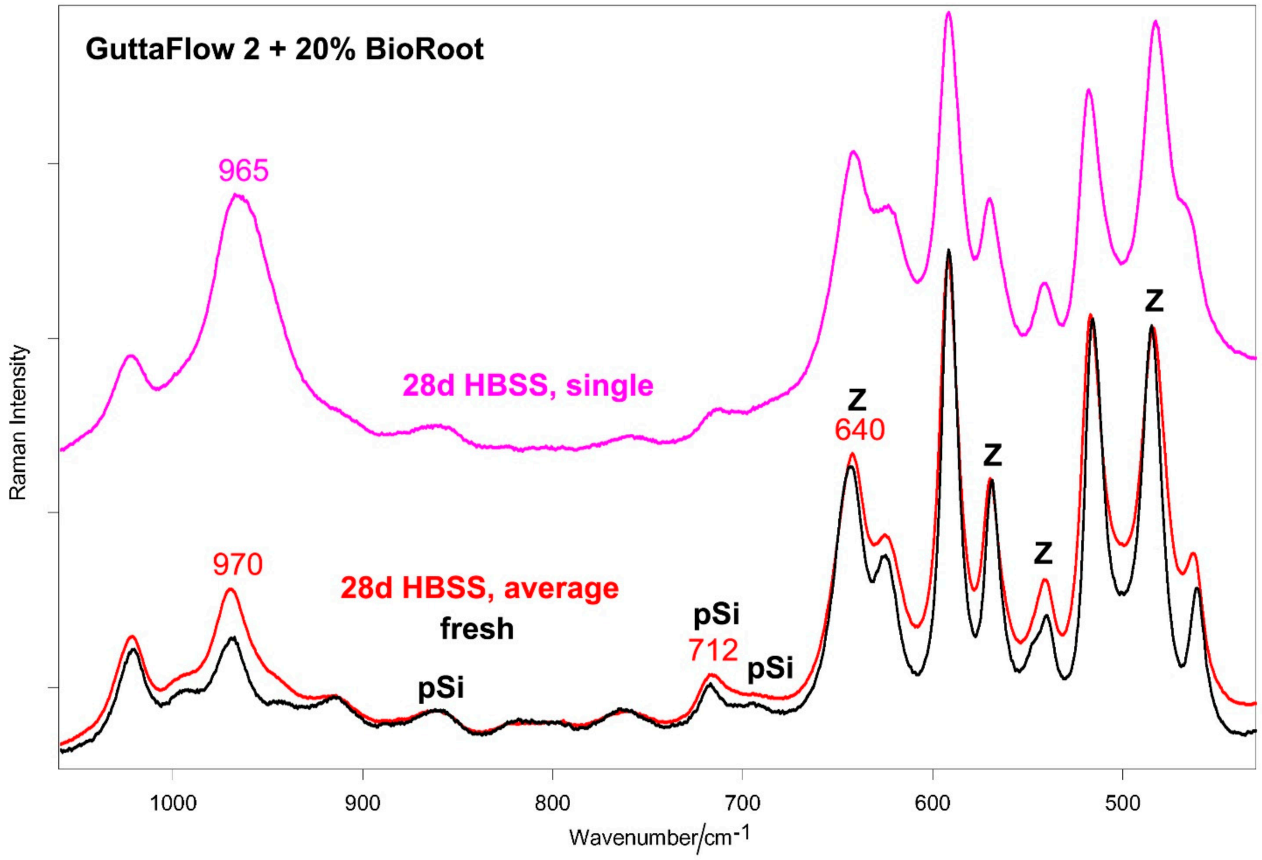


**Figure S19.** Average micro-Raman spectra recorded on the surface of GuttaFlow 2 + 20% BioRoot RCS, before (i.e. fresh) and after ageing in HBSS for 28 days. The spectra are normalized to the intensity of the 638 cm$^{-1}$ band. A single spectrum is shown as well (i.e. that recorded in the position that among those analysed revealed the thickest deposit). The bands assignable to monoclinic zirconia (Z) and polydimethylsiloxane (pSi) are indicated.

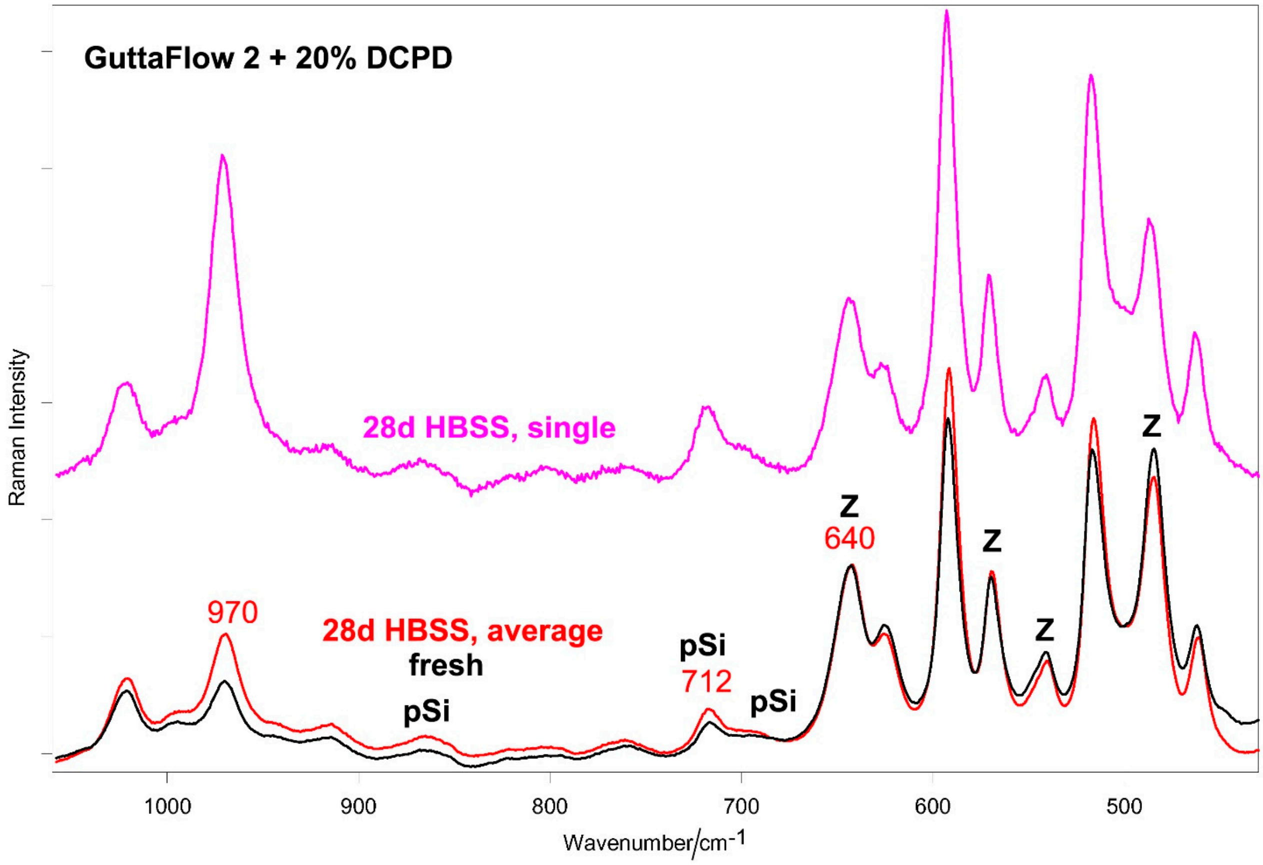


**Figure S20.** Average micro-Raman spectra recorded on the surface of GuttaFlow 2 + 20% DCPD, before (i.e. fresh) and after ageing in HBSS for 28 days. The spectra are normalized to the intensity of the 638 $cm^{-1}$ band. A single spectrum is shown as well (i.e. that recorded in the position that among those analysed revealed the thickest deposit). The bands assignable to monoclinic zirconia (Z) and polydimethylsiloxane (pSi) are indicated.

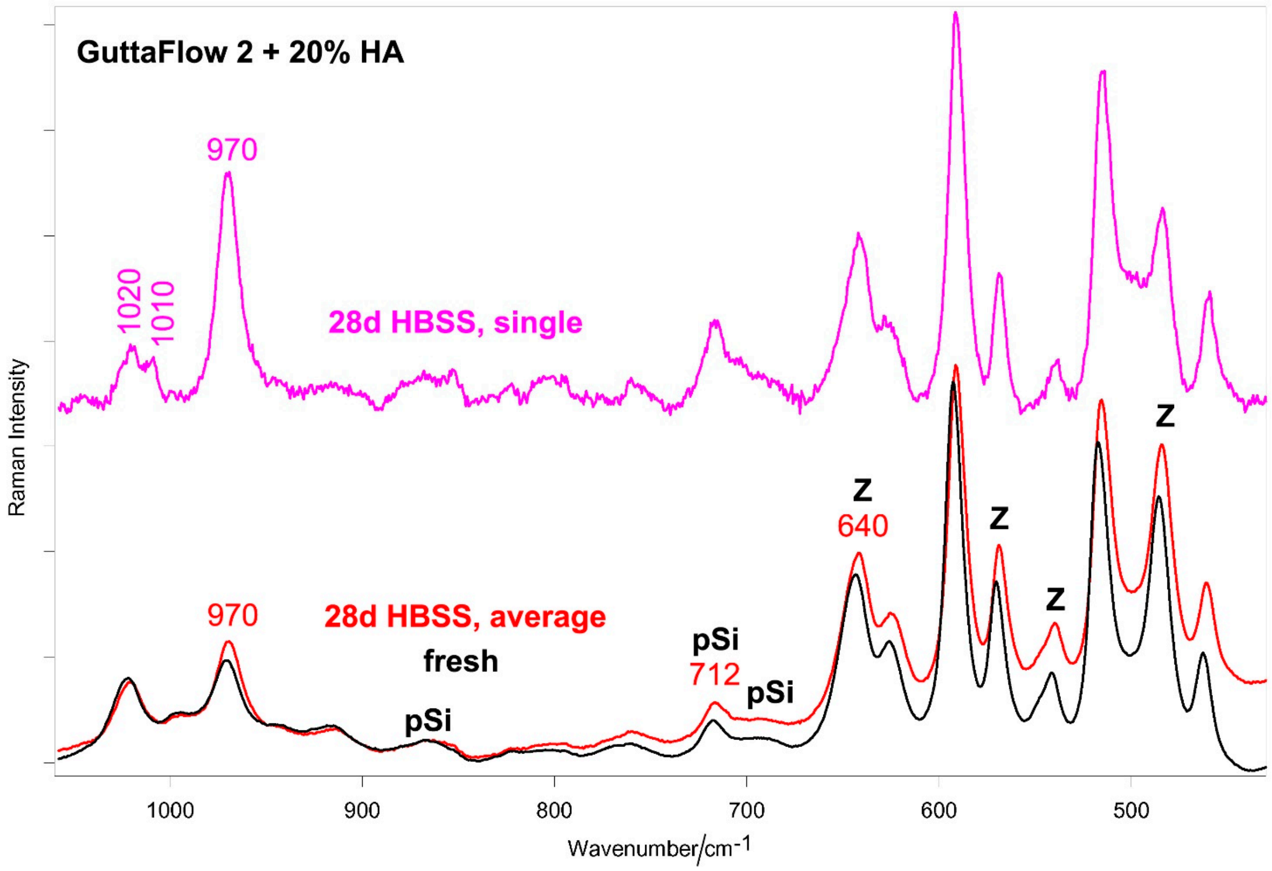


**Figure S21.** Average micro-Raman spectra recorded on the surface of GuttaFlow 2 + 20% HA, before (i.e. fresh) and after ageing in HBSS for 28 days. The spectra are normalized to the intensity of the 638 cm$^{-1}$ band. A single spectrum is shown as well (i.e. that recorded in the position that among those analysed revealed the thickest deposit). The bands assignable to monoclinic zirconia (Z) and polydimethylsiloxane (pSi) are indicated.
